# Poly(vinyl chloride) Plastisol Composites with Surface-Modified Wood Flour as Potential Coating and Insulating Materials for Modern Energy-Efficient Constructions

**DOI:** 10.3390/ma19010041

**Published:** 2025-12-22

**Authors:** Przemysław Siekierka, Edwin Makarewicz, Sławomir Wilczewski, Katarzyna Skórczewska, Krzysztof Lewandowski, Jacek Mirowski, Magdalena Osial

**Affiliations:** 1 Faculty of Chemical Technology and Engineering, Bydgoszcz University of Technology, 3 Seminaryjna Street, 85-326 Bydgoszcz, Poland; przemyslaw.siekierka@pbs.edu.pl (P.S.); edwin.makarewicz@pbs.edu.pl (E.M.); krzysztof.lewandowski@pbs.edu.pl (K.L.); jacek.mirowski@pbs.edu.pl (J.M.); 2 Institute of Fundamental Technological Research, Polish Academy of Sciences, 5B Pawińskiego Street, 02-106 Warsaw, Poland; mosial@ippt.pan.pl

**Keywords:** PVC plastisol, wood flour, wood–polymer composites, silane modification, eco-friendly insulation materials, coating composites, thermal stability

## Abstract

This study investigates the development of sustainable PVC-based composites filled with surface-modified wood flour for potential use in modern, energy-efficient building systems. The aim was to enhance the mechanical performance, thermal stability, and interfacial compatibility of PVC plastisols by incorporating fine- and coarse-grained coniferous wood flour modified with silane and surfactants. Composites were formulated using emulsion PVC (Vinnolit E-2059), bis(2-ethylhexyl) adipate as a plasticizer, and MARK-17 MOK as a thermal stabilizer, and were gelled under pressure at 150 °C. Their physical, mechanical, structural, and thermal characteristics were evaluated using density and hardness measurements, SEM, thermomechanical analysis, DMA, and TGA. The results demonstrated that composites containing fine-grained, silane-treated wood flour (Lignocel C-120) exhibited the most advantageous balance of stiffness, elasticity, and thermal resistance, attributable to improved polymer–wood interfacial adhesion. The findings confirm the potential of modified wood flour as an effective bio-based filler enabling the design of durable, thermally stable coating and insulating materials with reduced environmental impact. The proposed composites may serve as protective, bonding, or insulating layers in sustainable construction, supporting the development of innovative, wood-based materials for low-carbon building applications.

## 1. Introduction

Current climate and environmental policies increasingly promote low-carbon and circular construction strategies, including the use of renewable resources, waste valorization, and materials with favorable life-cycle performance. In this context, bio-based composites and wood-derived materials are regarded as promising solutions, as they can store biogenic carbon, reduce dependence on fossil resources, and enable lightweight structures with lower environmental footprints compared to conventional building materials [1,2].

Wood-polymer composites (WPCs), which combine the aesthetics of wood with the durability of polymers, are increasingly used in the construction industry. By incorporating lignocellulosic fillers into polymer matrices, WPCs combine the processability and weatherability of plastics with the renewable character and stiffness of wood, and are therefore increasingly considered as alternatives to solid wood and purely mineral-based solutions in energy-efficient construction [3]. They are used, among other applications, as decking boards, façade cladding boards, picket and panel fences, pergolas, and acoustic screens. WPC is also used in small garden architecture, for example, in the production of benches, planters, footbridges, and other outdoor elements.

Their growing popularity stems primarily from the advantageous properties of WPC, such as low water absorption, reduced susceptibility to cracking, good weathering resistance, and minimal maintenance requirements—they do not require painting, varnishing, or oiling. Additionally, these materials are highly resistant to decay and easy to shape, enabling the production of complex geometries. An essential advantage of WPC is the ability to incorporate recycled materials, which not only lowers production costs but also supports broader sustainability goals.

Poly(vinyl chloride) (PVC) is one of the most widely used polymers in the construction sector due to its high stiffness, dimensional stability, weather resistance, and ease of extrusion into complex profiles. For decades, rigid PVC-based composites have been applied in window frames, façade panels, siding, door profiles, pipes, and cable ducts, where long-term durability, impact strength, and low maintenance are essential. Among many polymeric matrices, PVC is attractive for WPC due to its properties. Studies have shown that lignocellulosic fillers incorporated into rigid PVC can enhance stiffness, reduce density, and improve the sustainability profile of rigid PVC products by lowering their fossil-based content. These composites align with current trends in sustainable construction, where materials that combine durability, recyclability, and a reduced carbon footprint are highly sought after.

However, most available research focuses on melt-processed rigid PVC–wood composites manufactured via extrusion or injection molding and used mainly for structural or semi-structural building components [4,5]. In contrast, information on PVC plastisols incorporating wood flour is minimal. Only recently, a study on PVC plastisol–wood flour composites was published, demonstrating that such systems can yield functional films, coatings, and insulating layers [6]. Plastisols—dispersion systems consisting of fine PVC particles suspended in plasticizers—enable the formation of flexible films, coatings, sealing layers, and bonding interfaces, which are essential in modern building envelopes and energy-efficient construction systems. Another 2024 study on plasticised PVC/wood composites highlights some processing and mechanical aspects but also shows the need for deeper investigation [7]. Yet, despite the growing interest in functional wood-based materials, only a small number of studies address the formulation, gelation behavior, interfacial adhesion, or performance of PVC plastisol–wood composites, and even fewer investigate the role of surface-modified wood flour in such systems. This gap is particularly notable given that plastisol processing differs fundamentally from melt compounding, and the interactions between plastisol-gelled PVC and lignocellulosic fillers remain poorly understood.

The key challenge in designing high-performance wood–polymer composites is the inherently poor compatibility between the hydrophilic, polar lignocellulosic phase and the typically hydrophobic, non-polar polymer phase. This mismatch in polarity results in high interfacial tension, incomplete wetting of wood particles by the molten polymer, and weak stress transfer across the interface, which manifests as reduced strength, toughness, and moisture resistance. Compatibilization strategies aim to overcome these limitations by creating chemical or physical bridges at the wood–polymer interface, reducing surface polarity differences, and improving wetting. This can be achieved by modifying the wood surface (e.g., with coupling agents or grafted polymers), by introducing reactive compatibilizers into the polymer matrix, or by simultaneously tailoring both phases. Recent reviews highlight that optimized interfacial engineering is crucial for maximizing the reinforcing efficiency of wood flour, achieving stable microstructures, and enhancing the mechanical and durability performance of WPCs in demanding applications such as building and construction [8].

Unmodified wood flour and fibers exhibit high hydrophilicity and limited interfacial compatibility with most hydrophobic thermoplastics, which typically leads to poor dispersion, weak adhesion, and reduced long-term durability of WPCs [9]. Therefore, numerous studies have focused on chemical or physical modification of wood flour to tailor its surface chemistry and improve interactions with the surrounding polymer matrix. Reported strategies include alkaline or oxidative treatments, acetylation, grafting of functional monomers, and the use of coupling agents or compatibilizers such as maleic-anhydride-grafted polymers, isocyanates, organosilanes, and other reactive oligomers [10,11,12]. These modifications have been shown to increase mechanical strength, reduce water uptake, and enhance dimensional stability in various wood-polymer systems, confirming that surface-engineered wood flour can act as an efficient reinforcing and functional filler in modern WPCs [13].

Among the various coupling and compatibilizing agents, organosilanes have attracted particular attention for natural-fiber and wood-based composites [9,14,15]. Trialkoxysilanes can hydrolyze and condense on the hydroxyl-rich wood surface, forming covalent or hydrogen-bonded networks that reduce hydrophilicity and introduce functional groups that interact with the polymer phase. Numerous studies have demonstrated that silane-treated wood flour or fibers exhibit improved interfacial adhesion, higher tensile and impact strength, and enhanced water and weather resistance in composites based on polypropylene, PLA, PVC, and other matrices [11,16,17].

Surfactants, on the other hand, are often used as auxiliary compatibilizers or dispersing agents that lower interfacial tension, promote wetting, and prevent agglomeration of wood particles during compounding [3,18].

Among many different polymers, poly(vinyl chloride) products are characterized by high mechanical strength and resistance to environmental and chemical factors. PVC plastisols can be obtained by mixing emulsion poly(vinyl chloride) with plasticizers, stabilizers, and other additives. In this study, a plastisol consisting of Vinnolit E-2059 emulsion PVC, with bis(2-ethylhexyl) adipate as the plasticizer and MARK-17 MOK as the stabilizer, was used for the tests. The polymer-wood composite was obtained by mixing PVC plastisol with modified wood flour. The flours came from coniferous trees and were named Lignocel C-120 and Lignocel L-9. A previously published paper [6] reported results from tests on composites made from PVC plastisol and the aforementioned wood flours, without any surface pretreatment. In the present work, before adding the wood flour to the plastisol, it was surface-modified with (3-chloropropyl)trimethoxysilane (referred to as silane in the work) or the nonionic surfactant Triton X-100. The tests of the composite material consisted of determining its gelation process, establishing optimal parameters for forming plates (films) by pressing, and then selecting their physical, physicochemical, thermomechanical, and structural properties.

## 2. Experimental Methods

### 2.1. Materials

The polymer matrix in the proposed plastisol was a poly(vinyl chloride) emulsion with paste-forming properties, named Vinnolit E-2059, for which K = 59.0 (the number-average molecular weight is 45,000, and the weight-average molecular weight is 100,000), the reduced viscosity was 86.0 cm^3^/g, and the bulk density was 0.520 g/cm^3^. The PVC was manufactured by Zakłady Vinnolit GmbH Co. KG, Carl-Zeiss-Ring 25, Ismaning, Germany.

The plasticizer in the plastisol was bis(2-ethylhexyl) adipate called Ergoplast ADO with a molecular weight of 370 g/mol, a density of 0.924 g/cm^3^, and a viscosity of 13.0 mPas. Zakłady Boryszew S.A., Boryszew ERG Sochaczew Branch, Sochaczew, Poland, manufactured the plasticizer.

The thermal stabilizer was octylcyanate mercaptide with the symbol MARK-17 MOK, manufactured by Galata Chemicals GmbH in Lampertheim, Germany.

Two types of wood flour from coniferous trees were used, namely Lignocel C-120 and Lignocel L-9, which differ in texture. The fine-grained wood flour was Lignocel C-120, with an average particle size of 0.07 to 0.15 mm; the bulk density was 0.1814 g/cm3; the oil number was 3.8 cm3/100 g; and the plasticizer number was 3.8 cm^3^/100 g. In this case, a paste formed during the determination of the oil number and plasticizer number. The average particle size of coarse Lignocel L-9 flour ranged from 0.80 to 1.10 mm; the particle structure of the flour was cubic, the bulk density was 0.1115 g/cm^3^, the oil number was 6.0 cm^3^/100 g, and the plasticizer number was 5.76 cm^3^/100 g. During the determination of the oil number and plasticizer, no paste formed; instead, a moist powder composed of individual flour particles was obtained. The wood flour was produced by J. Rettenmaier and Söhne GmbH Co KG, Rosenberg, Germany.

The modifier was (3-chloropropyl)trimethoxysilane (silane), CAS-2530-87-2, p.a. reagent from Sigma-Aldrich (St. Louis, MO, USA), and nonionic surfactant Triton X-100, CAS-9002-93-1, a product from Sigma-Aldrich.

Oleic acid was a colorless oily liquid with a molecular weight of 282.46 g/mol, boiling point 228.0 °C (at a pressure of 20.0 hPa), CAS No. 112-80-1, product No. 75090, analytical standard, produced by Sigma-Aldrich.

### 2.2. Methodology

#### 2.2.1. Determination of the Bulk Density of Wood Flour

The bulk density of wood flour was determined in accordance with PN-EN ISO 60 [19]. The measurement consisted of pouring wood flour, previously weighed to an accuracy of 0.001 g, through a funnel into a 100 cm^3^ measuring cylinder until a cone was formed above it. The surface of the wood flour was smooth with a flat spatula and weighed again, followed by the same procedure three times to estimate the bulk density, which was calculated by dividing the mass of the flour by the volume of the measuring cylinder.

#### 2.2.2. Determination of the Oil Number and Plasticizer Number

The oil number of wood flour was determined in accordance with PN-EN ISO 787-5 [20]. The measurement consisted of weighing 20.0 g of dried wood flour and placing it in a porcelain mortar. Four or five drops of oleic acid p.a. were added from a burette. After each portion of drops, the mortar contents were thoroughly ground with a porcelain pestle. During mixing, agglomerates of moist flour particles formed. From this point on, one drop of oleic acid was added, and the mixture was ground until a homogeneous mixture was obtained. The volume of oleic acid added was read to an accuracy of 0.1 cm^3^, while the oil number was expressed in cm^3^ of oleic acid per 100 g of wood flour. The plasticizer number was determined in the same way as the oil number. In this case, bis(2-ethylhexyl) adipate was used instead of oleic acid.

#### 2.2.3. Preparation of PVC Plastisol

The plastisol consisted of 900.0 g of powdered PVC polymer, 1080.0 g of ADO plasticizer, and 18.0 g of MARK-17 MOK stabilizer. All weighed plastisol components were placed in a mortar. The plastisol was mixed for approximately 8 h a day, with approximately 15-min breaks after each hour of grinding. The rest of the day, the plastisol was kept under vacuum. The plastisol was deaerated at rest in an HZV vacuum dryer at 6.5 hPa and 22 °C. Plastisol was tested 30 days after the end of the kneading and deaeration cycle. The plastisol samples were homogeneous, did not delaminate, and did not contain sediment. The exact method of plastisol production is given in monographs [6,21].

#### 2.2.4. Studies on the Gelation of PVC Plastisol and Its Composites with Wood Flour

A Brabender plastograph (Plasti-Corder PI 2200-3, Brabender GmbH and Co. KG, Duisburg, Germany) was used to evaluate the processing properties of PVC plastisol and plastisol composite with wood flour. The device was equipped with a thermostatic mixing chamber and two sigma rotors. The mixing chamber had a figure-eight cross-section and a capacity of 52.0 cm^3^. The rotors rotated in opposite directions at a speed ratio of 3:2. In the upper part, the chamber was connected to a loading hopper, which was closed with a flat pin. Sixteen samples were selected for testing, each containing a constant amount of plastisol (80.0% by weight) and a content of wood flour together with the modifier (20.0% by weight). The modifiers in the wood-flour mixture were 5, 10, 15, and 20% by weight in individual samples. The compositions of the individual samples were as follows: 1-(56.0 g PVC plastisol, 13.3 g wood flour, 0.7 g modifier), 2-(56.0 g PVC plastisol, 12.6 g wood flour, 1.4 g modifier), 3-(56.0 g PVC plastisol, 11.9 g wood flour, 2.1 g modifier), 4-(56.0 g PVC plastisol, 11.2 g wood flour, 2.8 g modifier). The wood flour used was Lignocel C-120 and L-9, and the modifiers were silane and Triton X-100. First, a mixture of wood flour and a modifier was prepared and then added to the plastisol. The preparation of wood flour modified with a silane or a surfactant involved thoroughly mixing both components in a mortar, followed by the addition of plastisol to the resulting homogeneous mixture. The test measured the torque of the faster of the two rotors, depending on the temperature in the mixer chamber. A constant number of rotor revolutions and the same temperature increase of 5 °C/min. The mixing resistance was measured dynamically on the motor shaft by rotating the rotors and recorded by a computer. The test results were presented as the relationship between torque and temperature.

#### 2.2.5. Production of Films from PVC Plastisol and Its Composites with Wood Flour

The composite was prepared by weighing samples containing a fixed amount of plastisol and a mixture of wood flour with a modifier in porcelain mortars. The individual samples contained: 1—40.0 g of plastisol, 9.5 g of flour, and 0.5 g of modifier, 2—40.0 g of plastisol, 9.0 g of flour, and 1.0 g of modifier, 3—40.0 g of plastisol, 8.5 g of flour, and 1.5 g of modifier, 4—40.0 g of plastisol, 8.0 g of flour, and 2.0 g of modifier. The contents of each mortar were thoroughly mixed and ground for 15 min until a homogeneous, uniform mass was obtained. The resulting compositions were placed inside the template field. The template was made of brass and had a square frame shape, with external dimensions of 150 × 150 mm, internal dimensions of 120 × 120 mm, and a thickness of 2.0 mm. The template was placed on a Teflon film applied to a polished steel plate. A metal spatula was used to spread the plastisol composition with wood flour inside the template and level the surface. A Teflon film and a polished steel plate were applied. The steel sheet had the template’s external dimensions and a thickness of 2.0 mm. The sample prepared in this way was placed in a hydraulic press for gelation. The gelation process consisted of compressing the sample to 1.5 N/cm^2^ and holding for 3.0 min, then increasing the pressure to 18.0 N/cm^2^ and holding for another 3.0 min. After gelation was complete, the pressure was released, the sample was removed from the hydraulic press, and cooled. The composites were gelated at 150 °C. In this way, membranes were obtained for further testing.

#### 2.2.6. Density Measurements

The density of films obtained by gelation of PVC plastisol at different temperatures and of polymer-wood composites was determined by the pycnometric method using a Pycnomatic and a Pycnomatic ATC device from Thermo Fisher Scientific, Waltham, MA, USA. This device uses gas displacement to determine the actual density. Helium was used as the gas because helium atoms are tiny and can penetrate even the microscopic pores of the tested material. This enabled the determination of the actual volume occupied by the tested sample. The ratio of the sample’s mass to its actual volume was the material’s density. The measurement was performed in accordance with PN-EN ISO 1183-3 [22].

#### 2.2.7. Determination of the Hardness Using the Shore Method

The hardness of plastisol films or their composites with wood flour was measured using a Shore A hardness tester (Zwick Roell, Ulm, Germany) in accordance with the PN-EN 868-2003 [23] standard. The test samples consisted of two films joined together and were 4.0 mm thick. The device was calibrated after each measurement. The hardness value of the material was read after 30 s, when it had stabilized. The result was taken as the average value of three hardness measurements at different points on the film.

#### 2.2.8. Morphology Studies

The morphology was studied using a Zeiss Crossbeam 350 scanning electron microscope (SEM) (Zeiss GmbH, Oberkochen, Germany). Cryogenic fractures of composite samples were obtained by cooling them in liquid nitrogen and then breaking them. The fractures were observed with a gold coating.

#### 2.2.9. Consistometric Tests

Thermomechanical tests were performed using a Höppler consistometer manufactured by VEB MLW Prűfgerate-Werk Medingen/Sitz Freital, Freital, Germany. The total load on the membrane sample was 494 g. The measurement was performed in the temperature range from 20 to 115 °C. The temperature rise rate was approximately. 2.0 °C/min. The test results are presented as the relationship between the membrane sample’s deformation under load and temperature. The relationships obtained were described using the Excel software package.

#### 2.2.10. Mechanical Tests

Tensile strength tests on the samples were performed on an Instron 5966 testing machine (Zwick Roell, Ulm, Germany) at a stretching speed of 30.0 mm/min. For strength testing of composite films containing 20% by weight of modified wood flour, strips measuring 110.0 × 10.0 mm were cut. The length and width of the part attaching the strips to the strength testing machine holder were 10.0 × 15.0 mm. Five tear tests were performed for each membrane. The average value was taken as the result.

#### 2.2.11. DMA Tests

Tests to determine the elastic modulus (E’), loss (E”), and mechanical loss factor (tan δ) were performed using a Netzsch DMA 242D analyzer (NETZSCH, Selb, Germany) equipped with a three-point bending attachment with a support spacing of 20 mm. A sample measuring approx. 40.0 × 5.0 × 2.5 mm was bent by 20 µm at 1 Hz over a temperature range from −175 °C to 115 °C. The heating rate was 3 °C/min.

#### 2.2.12. Thermal Analysis

Thermogravimetric tests of plasticizer composite film samples with modified wood flour were performed using a TG 209 F3 Tarsus device manufactured by Netzsch, Selb, Germany. They enabled the determination of phase transition temperatures occurring in one or two stages of thermal decomposition of the tested materials. The initial decomposition temperatures, the temperature corresponding to the first or second decomposition maximum, and the final decomposition temperatures were determined. The mass losses of the tested samples during the first and second stages of decomposition were determined. The detailed procedure for preparing the samples for testing and performing the tests is described in ASTM E1356-08 [24]. The PVC used decomposed in the range of 268 °C to 333 °C, the ADO plasticizer in the range of 240 °C to 278 °C, and wood flour in the range of 317 °C to 380 °C. References [25,26,27,28] were used to interpret the test results.

#### 2.2.13. Determination of the Leaching from Membranes

The method for determining the content of substances that were not fully bound to the membrane relied on the ability of water at 100 °C to dissolve or emulsify them. In this way, the amount of substances removed from the membrane could be quantified. The determination consisted of preparing a sample weighing approximately 20 g of membrane sample, weighing it on an analytical balance with an accuracy of 0.001 g, and then tightly wrapping it in filter paper. All samples of the tested membranes were prepared in this way and then placed in boiling distilled water for two hours. A 1.0 dm^3^ beaker containing 800 cm^3^ of water was used. Eight membrane samples were placed in the beaker at a time. After the specified time, the membrane samples were removed from the water, cooled in the air, and unwrapped from the filter paper. The samples were then rinsed in distilled water and dried in a forced-air dryer at 60 °C until a constant weight was achieved. The difference in weight between the membrane samples before and after leaching in boiling water was used to calculate their weight loss. In this study, we use the percentage reduction in membrane weight.

## 3. Results

**Figure 1:**
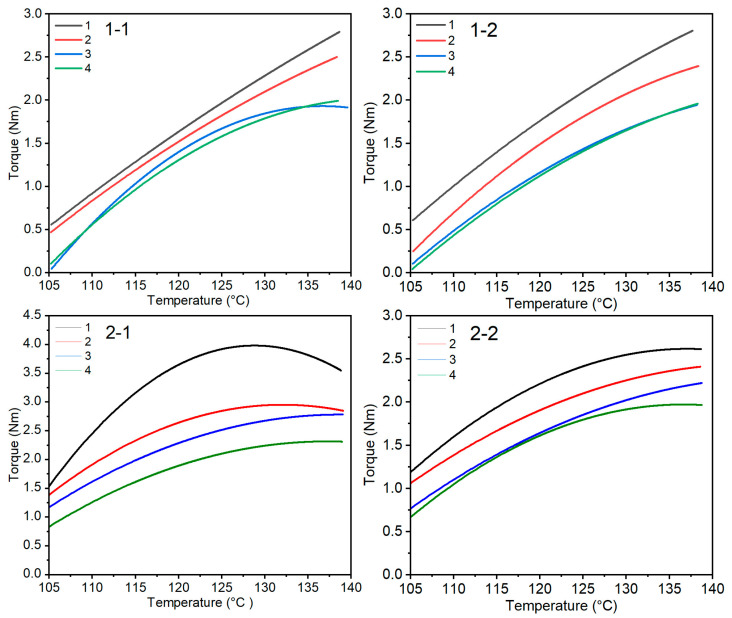
Gelation curves of PVC plastisol compositions with modified wood flour. Explanations: 1-1 PVC plastisol composition with Lignocel C-120 flour and silane, 1-2 plastisol composition with Lignocel C-120 flour and Triton X-100, 2-1 PVC plastisol composition with Lignocel L-9 flour and silane, 2-2 plastisol composition with Lignocel L-9 flour and Triton X-100, 1—5.0% by weight, 2—10.0% by weight, 3—15.0% by weight, and 4—20.0% by weight of modifier in wood flour.

The gelation of PVC plastisol compositions with modified wood flour was tested using a Brabender plastograph. Based on data recorded by the computer, drawings and descriptions of the gelation process for individual compositions were prepared. Figure 1 shows the torque-temperature curves for all tested samples. They were used to determine the temperature at which the torque of the rotors reached a maximum.

The tests showed that the type of wood flour and the type of modifier used had a decisive influence on the gelation process of the PVC plastisol composition with wood flour. Under the influence of increasing temperature, the plastisol composition with the modifier underwent gelation. This consisted of the plasticization of emulsion poly(vinyl chloride) grains, leading to an increase in the material’s viscosity. On the other hand, the relationship between the change in torque and increasing temperature was used to describe the gelation process within Equation (1):

M = −aT^2^ + bT − c (1)

where the following applies:

M is the torque in Nm, T is the temperature measured in the crumple chamber in °C, and a, b, and c stand for constants.

The constant (a) is associated with the pseudoplastic flow of the material caused by the strong absorption of the plasticizer by the surface of the wood flour particles. The constant (b) is associated with Newtonian flow behavior resulting from the wetting of the wood flour particle surfaces by the plastisol. The value of the constant (c) takes into account the size of the wood flour particles. Table 1 shows the values of the constants in Equation (1) and the determined gelation temperatures at maximum torque.

Table 1 presents the results of the gelation test of plastisol composite with modified wood flour. In the case of plastisol composite with Lignocel C-120 flour containing silane or Triton X-100, the maximum gelation temperatures were similar and differed only slightly from each other. Increasing the amount of silane or Triton X-100 in the composition resulted in a significant decrease in torque. In turn, in the plastisol composition with modified Lignocel L-9 flour, we observed that, initially, with increasing amounts of silane or Triton X-100 in the tested system, the maximum gelation temperature increased slightly, then decreased. In the case of these compositions, the torque values decreased. Equation (1) describing the gelation of the plastisol composite showed that it was a polynomial function. This description of the gelation phenomenon of the plastisol composition was influenced by many factors resulting from the structure of the system itself and the properties of its individual components. Most likely, the values of constants “a” and “b” (Table 1) characterize the occurrence of interactions in plastisol between polymer macromolecules and the surface of wood flour particles. Their higher value indicated a stronger bond between the surface of the flour particles and the plastisol. Another factor could have been the size of the wood flour particles. The largest particles were those of Lignocel L-9. In the case of the composition in which it was present, associations consisting of many individual particles could have formed. This was evidenced by the highest value of the constant “a” (Table 1). The porous structure of the flour particles also influenced the values of the constants discussed. In this case, the Lignocel C-120 flour particles were more porous. This was indicated by the lower value of the constant “c” compared to analogous compositions containing Lignocel C-120 (Table 1). Overall, the study showed that silane was a better compound for increasing the bonding of flour particles with plastisol than Triton X-100. However, it can be concluded that the modifier in wood flour should not exceed 15.0% by weight. A higher amount of modifier in the flour resulted in lower torque and a lower gelation temperature, which may adversely affect the product’s properties.

PVC plastisol composites with modified wood flour were produced using a constant temperature pressing method in the form of plates or films from which samples were cut for testing. Table 2 presents the results of density and hardness tests of the produced polymer composites.

The data presented in Table 2 show that the density of films made of PVC plastisol with the addition of Lignocel C-120 wood flour containing silane was slightly higher than that of films containing Triton X-100. However, for Lignocel L-9 wood flour, we found similar densities in films containing either silane or Triton X-100. In all cases of the tested films, the increasing amount of silane or Triton X-100 in wood flour did not affect their density. The slight differences in the density values of the polymer composites produced, depending on the amount of silane or Triton X-100 added, could have been caused by the irregular shape and size of the flour particles, as well as their uneven distribution within the membrane, rather than by the amount or type of modifier. There may also have been uneven absorption of modifier molecules by the porous surface of the flour particles into their interior. Hence, it can be concluded that composites made with Lignocel C-120 flour had a slightly higher density than composites made with Lignocel L-9 flour. The Shore method was used to measure the hardness of composite membranes with modified wood flour. The following columns of Table 2 show the results of testing their hardness before and after leaching in boiling water. The tests showed that a higher content of the modifier in the flour resulted in a decrease in its hardness. The composite material’s hardness increased significantly when Lignocel L-9 flour was used in its production. Most likely, in this case, it was also related to the composite membrane’s structure, which was influenced by the size and hardness of the flour particles. In turn, boiling water leaching tests of the polymer composite samples showed that when Lignocel C-120 wood flour and silane were used, the amount of leached substances increased with the amount of silane in the flour. In the case of the other composite samples tested, it can be concluded that the highest amount of substances is leached from materials containing Triton X-100, regardless of the type of wood flour used. The results obtained indicate the probable leaching of both the modifying additive and the plasticizer from the membrane samples. Repeated tests of the hardness of the films after leaching showed that in the case of Lignocel C-120 wood flour, their hardness increased. In contrast, the hardness of films containing Lignocel L-9 wood flour decreased significantly. The decrease in membrane hardness is greater the more wood flour-modifying additive the composite contains. After leaching, a reverse trend in hardness was observed for the plastisol composite with Lignocel L-9 flour and a high proportion of the modifying additive. As a result, its leaching out reduced the interaction of polymer macromolecules with the filler surface.

**Figure 2:**
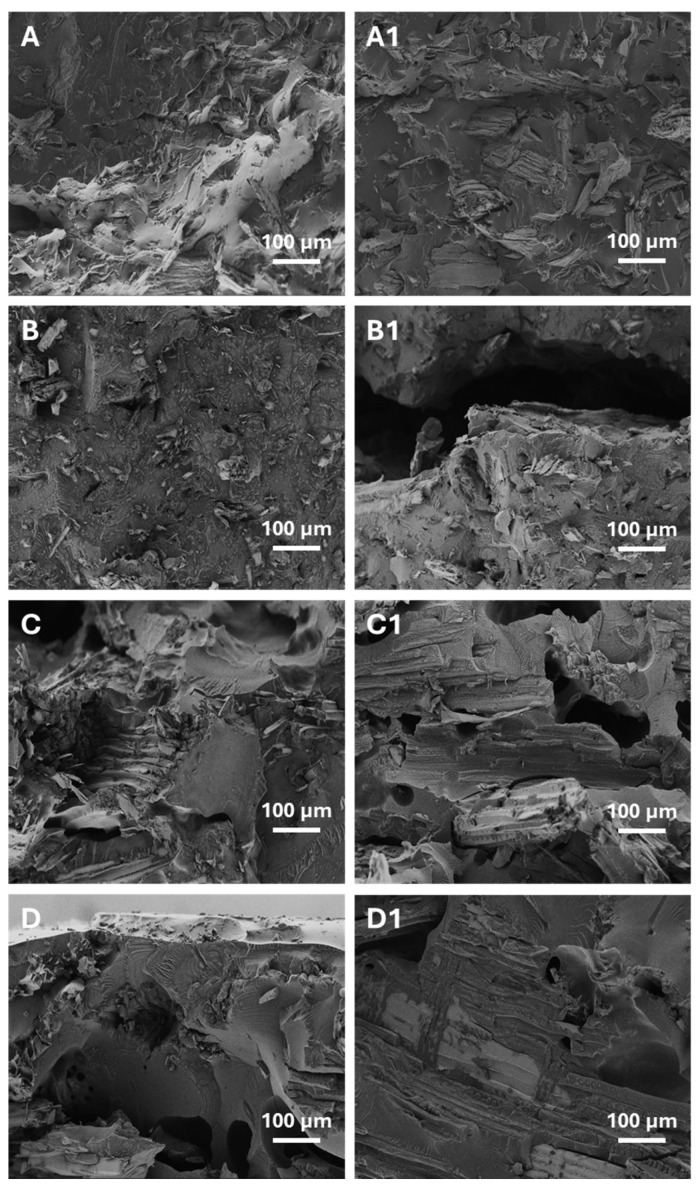
SEM images of the cross-section of the membrane containing modified wood flour before and after leaching in boiling water. (**A**,**C**)—membrane samples with Lignocel C-120 flour containing 20% by weight of silane. (**B**,**D**)—membrane samples with Lignocel L-9 flour containing 20% by weight of Triton X-100. (**A1**–**D1**)—membrane samples after leaching in boiling water.

The gelation of PVC plastisols containing wood flour modified with silane or Triton X-100 on the surface led to different results, indicating an ambiguous gelation process and composite structure. The effect was clearly confirmed by membrane density tests and, above all, by hardness measurements of membrane samples before and after boiling-water leaching. Figure 2 shows enlarged fragments of breaks in selected membrane samples containing modified wood flour before and after leaching in boiling water.

The images of the membrane cross-sections shown in Figure 2 revealed significant differences between samples containing different fillers and between samples before and after boiling in water. The membrane samples marked A and C contained fine-grained wood flour, while B and D contained coarse-grained wood flour. The images clearly showed the differences in their sizes. However, the modifiers themselves were not clearly seen. The effects of boiling water on the membrane containing the wood flour modifier were clearly visible. In this case, the most severe damage to the membranes was caused by boiling water, resulting in deep, empty spaces (called caverns), as shown in images B1 and D1. These membrane samples contained Triton X-100, a nonionic surfactant that is easily soluble in water. In contrast, the use of silane to modify fine-grained wood flour resulted in minimal leaching. The observed effect is confirmed by the data presented in Table 2 and image A1. On the other hand, the modification of coarse wood flour with silane was less effective. Image C1 shows areas of silane loss in the form of small pores distributed within the structure of the material.

**Figure 3:**
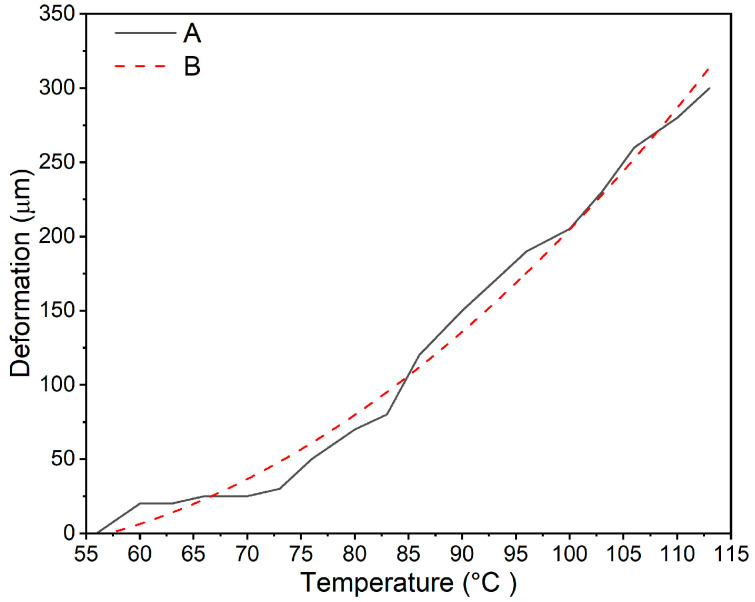
Temperature dependence of deformation for a composite membrane consisting of plastisol and Lignocel C-120 powder containing 10% by weight of Triton X-100 surfactant. A—actual course of the dependence, B—interpolated course described by a polynomial.

Next, thermomechanical tests of plasticizer-composite film samples containing modified wood flour were performed using a Höppler consistometer. The measurement consisted of recording the sample’s deformation, known as deformation from temperature under constant load. Figure 3 shows a typical curve for the deformation as a function of temperature for the tested material.

The course of the deformation-temperature relationship shown in Figure 3 could be described using a general polynomial formula in the form:

D = dT^2^ + eT − f (2)

where D is the deformation in µm, and T is the temperature in °C, while d, e, and f are the constants of the equation.

Table 3 shows the values of the calculated constants in Equation (2) for all tested compositions.

The data in Table 4 show that, in all cases of PVC plastisol composites with wood flour and a modifying additive, a parabolic relationship was observed. Its shape was essential because it changed significantly as the amount of modifying additive in the composition increased. The analysis of the constant “a” shows that its decreasing value indicates a change in its shape towards a curve with less concavity. This meant that as the amount of the modifying additive in the composite increased, its shape tended to become straighter. This phenomenon is confirmed by the increasing values of constants “b” and “c” and the decreasing value of constant “a.” In fact, during deformation in the membrane, the forces binding the surface of wood flour particles to polymer macromolecules weakened, and this weakening was greater the more modifier there was in the composite. In composites containing Lignocel L-9 flour, the weakening of the interaction between the flour particles and the polymer was slightly greater than in the case of Lignocel C-120 flour.

**Figure 4:**
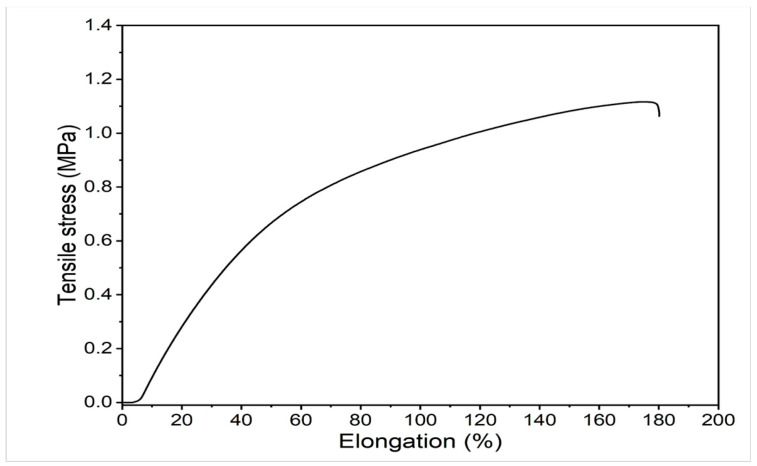
Tensile stress as a function of relative elongation for a PVC plastisol polymer composite with Lignocel C-120 flour containing 15% by weight of silane.

Products made of polymer-wood composites can be used in a wide variety of environmental conditions. Among other things, they can be subjected to significant static and dynamic mechanical forces. For example, they may be subjected to tearing, which can be characterized by determining the maximum tensile stress at break and the corresponding relative elongation. Figure 4 shows a typical curve obtained during the tearing of a sample of one of the membranes of the tested composite materials. In contrast, Table 4 presents the results of the mechanical strength test for the tearing of polymer composites containing modified wood flour.

The data presented in Table 4 show that the highest values of maximum tensile strength and relative elongation were obtained for membranes made of a composite containing Lignocel C-120 flour and silane. Next in line was the composite with Lignocel C-120 flour and Triton X-100. In this case, the maximum tensile stress was approximately 25% lower, and the relative elongation was almost twice as low. In the third and fourth cases, the values of maximum tensile strength and relative elongation were similar, although much lower than in the case of composites with Lignocel C-120 flour. In the case of all membranes, after leaching, there was a decrease in the maximum tensile strength, while the relative elongation increased or remained virtually unchanged. The more modifiers there were in the composite, the smaller the changes were. The tests showed that the strength properties of polymer-wood composites are influenced by the type of flour, with Lignocel C-120 and a modifying additive such as silane proving to be the best. Composites containing Lignocel L-9 flour exhibit poorer properties, resulting in the break at very low values of maximum stress and relative elongation.

**Figure 5:**
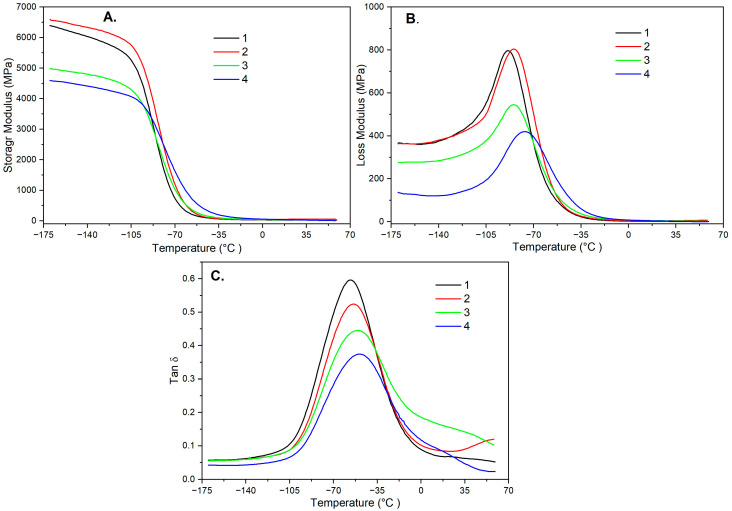
Relationship between the Storage modulus (**A**), loss modulus (**B**), Tangent δ (**C**), and temperature for compositions consisting of plastisol and Lignocel C-120 flour with 20 wt% by weight of silane (1) or Triton X-100 (2), and plastisol with Lignocel L-9 flour with 20% by weight of silane (3) or Triton X-100 (4).

One of the important tests characterizing construction materials was to determine their properties during the transition from a glassy state through a highly elastic state to a viscous state. The tests were performed using dynamic mechanical thermal analysis. Figure 5A–C shows the relationships between the change in the conservative modulus, also known as the elastic modulus, and the loss and mechanical loss coefficient as a function of temperature. Table 5 and Table 6 contain data on the determined values of the modules and the mechanical loss coefficient depending on the wood flour and modifier used.

The mechanical properties of composites with modified wood flour were closely related to their structure. As a result of the tests, data were obtained determining the influence of their composition on the mechanical behavior of the tested material. Table 5 presents the determined initial, deflection, and final temperatures, along with the corresponding conservative modulus values. These showed that the introduction of larger amounts of silane or Triton X-100 into the polymer composition only slightly increased the initial temperature, which was in fact the glass transition temperature and determined the transition of the material from a glassy to a highly elastic state. At the same time, in the case of polymer compositions containing silane, the elastic modulus increased slightly, and in the case of Triton X-100, the value of this modulus remained virtually unchanged. In turn, the use of Lignocel C-120 flour, silane, and Triton X-100 increased the flexural temperature slightly. It determined the maximum properties of the material in a highly elastic state. In the case of composites with Lignocel L-9 flour and silane, the deflection temperatures remained virtually constant. For the same composites with Triton-X-100, the flexural temperature values increased slightly. It could be concluded that as the amount of silane or Triton X-100 in the polymer composition increased, the elastic modulus value also increased. The final temperature corresponded to the transition of the polymer composition from a highly elastic state to a viscous state and is practically impossible to determine. In compositions containing Lignocel C-120 flour and silane, the final temperatures remained almost constant. However, for the same compositions with only Triton X-100, the final temperatures increased slightly. The situation was similar for compositions with Lignocel L-9 flour and silane, for which the final temperatures were practically constant. However, for compositions with Triton X-100, the final temperatures increased slightly. The elastic modulus values for the compositions with Lignocel C-120 flour were zero. This meant that these compositions lacked elastic properties. On the other hand, the compositions with Lignocel L-9 flour showed practically insignificant constant elasticity. The obtained test results can be justified by the fact that in the case of Lignocel C-120 flour, the modifier is absorbed into the highly developed surface structure of its particles. In contrast, Lignocel L-9 flour particles are large and have a surface that the modifier cannot penetrate. Therefore, the composites produced with Lignocel L-9 flour exhibited lower elastic modulus values and higher phase transition temperatures. Table 6 presents the determined values of maximum temperature, loss modulus, maximum mechanical loss factor temperature, and mechanical loss factor.

The analysis of these data showed that the introduction of a larger amount of silane into the polymer composition with fine-grained wood flour resulted in the greatest reduction in maximum temperature, and in these cases, the highest loss modulus values were obtained. The changes were similar for the other composite films. The highest negative values of the temperature corresponding to the maximum value of the mechanical loss factor were determined for the film made of a plastisol composition with fine-grained wood flour and silane. Slightly lower values were obtained for compositions of the same flour with Triton X-100. The lowest negative values of the maximum temperature and the corresponding lowest values of the loss modulus were obtained for compositions with coarse wood flour and Triton X-100. They also corresponded to the highest values of temperature at the maximum temperature and the lowest values of the mechanical loss factor. It was found that as the amount of Triton X-100 in the polymer composition increased, the loss modulus decreased. In general, the test results can be explained by the free movement of polymer macromolecules relative to each other. This meant that most of the energy supplied to the composition was converted into work rather than heat. Therefore, composites with coarse flour exhibit lower loss modulus values.

**Figure 6:**
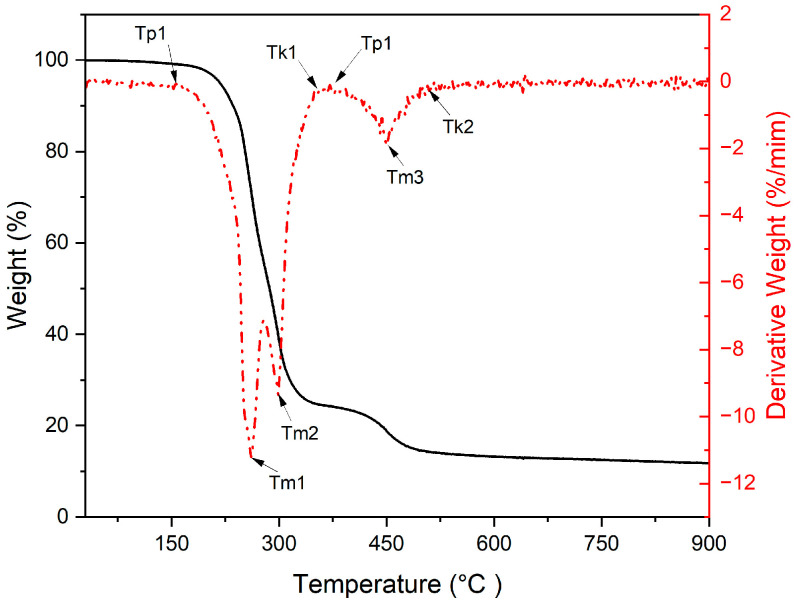
Differential thermogravimetric curve of the mass loss as a function of temperature for a membrane made of PVC plastisol and Lignocel C-120 wood flour containing 5.0% by weight of silane. Tp—initial decomposition temperature, Tm—inflection temperature, the highest decomposition rate, and Tk—final temperature.

Thermogravimetric studies of the films allowed the influence of temperature on their thermal decomposition process to be determined. Figure 6 shows a typical TGA curve for a composite film sample. For the other films, differential curves with a similar or very similar shape were obtained.

Table 7 presents the characteristic temperatures determined from the differential curves obtained from the thermal decomposition of the tested membranes.

The data presented in Table 7 show that in the case of membranes containing fine-grained wood flour and silane, the initial decomposition temperature increases with the amount of modifier in the composite. According to the same relationship, in the first stage of decomposition, the temperatures of the first and second maxima and the final temperatures increased. The relationships observed also applied in the second stage of decomposition. The calculations showed that in each case, approximately 90.0% by weight of the total mass of the film decomposed in the first stage. A comparison of the determined temperatures with the temperatures of individual plastisol components given in the test methodology showed that the initial decomposition temperatures refer to PVC. However, its main decomposition took place in the temperature range between the first and second maxima to the temperature ending the first stage of decomposition. In the second stage, the wood flour essentially decomposed, and secondary reactions took place between the resulting substances. It can be concluded that the addition of silane to wood flour increased the thermal stability of the films by raising the individual decomposition temperatures. In turn, thermogravimetric studies of membranes containing fine-grained wood flour modified with a nonionic surfactant showed that during their thermal decomposition, the initial decomposition temperature decreased with an increase in the amount of Triton X-100 in the composite. This meant that the presence of Triton X-100 in the membrane accelerated its decomposition and thus reduced its thermal resistance. The maximum and final decomposition temperatures of these membranes were also reduced. In the second stage of decomposition, the initial, maximum, and final decomposition temperatures were similar to those of the wood flour composite with silane, but they showed decreasing values. Based on the tests performed so far, it was found that the addition of Triton X-100 to fine-grained wood flour reduced the thermal resistance of the composite. When testing the third type of membranes consisting of plastisol and coarse wood flour with silane, it was found that among all the composite membranes presented in Table 7, their initial decomposition temperature had the highest values and increased with the amount of silane. The tested composites had only one maximum decomposition temperature, which was practically independent of the amount of silane in the film. The final temperature indicated a constant value independent of the amount of silane in the film. In the second stage of decomposition, however, we noticed a complete similarity to the first composite. The fourth composite membranes, consisting of plastisol and coarse wood flour with Triton X-100, showed a high similarity to the second composite membranes during thermal decomposition. In the first stage of decomposition, the initial, maximum, and final temperatures decreased with increasing amounts of Triton X-100 in the membranes. In contrast, the course of the second stage of thermal decomposition of the fourth composite membranes was analogous to that of the second stage of the second composite. Next, composite membrane samples were examined after leaching in boiling water. The results of thermogravimetric analysis are presented in Table 8.

The data presented in Table 8 showed that the TGA curves obtained in the first stage of membrane decomposition did not show a peak corresponding to the second maximum of decomposition (T_m2_). The removal of water-soluble substances from the membranes containing fine-grained Lignocel C-120 flour and silane caused the initial decomposition temperature to increase as the amount of silane in the flour increased, the maximum temperature to decrease, and the final temperature to remain virtually constant. In the second stage of membrane decomposition, the products formed showed increasing initial, maximum, and final decomposition temperatures. In turn, membranes containing the same flour but modified with Triton X-100 showed different behavior during thermogravimetric testing. As the amount of Triton X-100 in the flour increased, the initial and maximum decomposition temperatures decreased. The final temperature remained virtually unchanged. In the second stage of decomposition, we observe a decrease in the initial decomposition temperature and virtually constant values of the maximum and final decomposition temperatures. Comparing the decomposition temperatures of membranes modified with silane and Triton X-100, we conclude that the temperatures are the same or very similar. In turn, thermogravimetric studies of membranes containing coarse Lignocel L-9 flour and silane showed that in the first and second stages of decomposition, as the silane content in the flour increases, the initial decomposition temperature decreases. In contrast, the maximum and final temperatures remain virtually unchanged. A similar situation can be observed with membranes containing the same flour and Triton X-100. In this case, we observed slightly higher initial decomposition temperatures of the membranes in the second stage of their thermal degradation.

## 4. Discussion

Plastographic studies of plastisol compositions containing modified wood flours demonstrated that both the type and the amount of modifier significantly influenced the gelation process. For fine-grained flour modified with small quantities of silane or surfactant (5–10 wt%), the torque and maximum gelation temperature remained constant. Increasing the modifier content to 15–20 wt% decreased torque while maintaining a stable maximum temperature. For coarse wood flour, the maximum gelation temperature remained constant regardless of modifier type or concentration. In contrast, torque initially increased at low modifier concentrations and decreased at higher levels, consistent with observations for PVC-based wood–plastic composites in recent studies [6,7].

These results indicate that 15 wt% modifier was the most advantageous concentration, aligning with literature reporting optimal additive levels for balancing the mechanical and processing properties of PVC–wood composites [6].

Density measurements of PVC plastisol membranes showed that introducing 5–20 wt% of silane or surfactant had no meaningful effect on membrane density. Minor variations are likely due to the irregular particle geometry, polydispersity, and heterogeneous morphology of wood flour, which may lead to uneven modifier absorption and nonuniform distribution within the polymer matrix. Similar effects of particle morphology on composite structure and density have been widely described in recent reviews on wood–plastic composites (WPCs) [29].

Hardness tests performed before and after boiling revealed significant leaching of Triton X-100, regardless of flour type. After leaching, the hardness of composites with fine-grained flour increased, whereas that of composites with coarse flour decreased, with larger reductions corresponding to higher Triton X-100 content. The strong influence of water exposure and leaching on mechanical properties is consistent with earlier findings for PVC- and PP-based WPCs [7,30]. Fracture images confirmed more extensive leaching in coarse-flour composites modified with Triton X-100, while silane-modified membranes exhibited substantially lower leaching. The mitigating effect of silane treatments on water absorption and property degradation has been reported in multiple studies [30,31,32].

Thermomechanical testing demonstrated that deformation during loading was associated with weakening interactions between wood flour surfaces and polymer macromolecules. This effect increased with higher modifier content and was more pronounced in composites with coarse flour, likely due to deeper modifier penetration and greater adsorption. These findings agree with studies that found that DMA analysis showed that interfacial adhesion and filler mobility strongly influence viscoelastic behavior in WPCs [5,33].

Mechanical strength testing showed that membranes containing fine-grained flour modified with silane exhibited the highest tensile strength and elongation. Slightly lower values were obtained for fine flour modified with Triton X-100, whereas composites with coarse flour showed significantly poorer strength. Literature confirms that both particle size and surface treatment—particularly silanization—are key determinants of mechanical performance in wood–polymer composites [29,31]. After boiling, the mass and maximum tensile stress decreased for all samples, whereas elongation increased or remained unchanged. Fine-flour composites modified with silane showed the least mass loss and the best retention of mechanical properties, which agrees with reports indicating that effective coupling agents substantially reduce water-induced degradation [32].

DMA analysis revealed that silane in fine-flour composites produced the most significant decrease in transition temperature and the highest loss modulus values. Similar but less pronounced effects were observed in other systems. The most substantial negative shift in maximum temperature and the highest loss factor occurred in membranes containing fine flour with silane, followed by those containing fine flour with Triton X-100. The lowest values were found in composites containing coarse flour and Triton X-100. Increasing Triton X-100 content reduced the loss modulus in coarse-flour composites, suggesting greater particle mobility during thermal and mechanical loading, consistent with viscoelastic trends reported for WPCs [5].

Thermogravimetric analysis showed that composites with fine flour and silane exhibited improved thermal stability, whereas Triton X-100 reduced thermal resistance. Composites with coarse flour and silane demonstrated the highest overall thermal stability. After leaching in boiling, silane-modified membranes showed only slight deterioration, while Triton-modified membranes—after removal of water-soluble compounds—showed thermal behavior approaching that of silane-modified systems. These results are in line with recent findings on the thermal behavior of PVC-based wood composites containing different coupling agents [6,7].

## 5. Conclusions

The conducted research demonstrated that the physico-mechanical and thermal properties of PVC plastisol composites can be effectively improved by incorporating surface-modified wood flour and appropriately selected interfacial modifiers. The most favorable reinforcement effect was observed with fine-grained wood flour modified with silane, resulting in the highest tensile strength, good elongation at break, and enhanced thermal stability. In contrast, the use of Triton X-100 in composites containing coarse wood flour proved less effective, leading to increased susceptibility to deformation, significant mass loss after leaching, and a reduction in loss modulus due to greater mobility of filler particles.

It was also confirmed that efficient gelation of PVC plastisols occurs at wood-flour contents below 15 wt%, ensuring a uniform microstructure and stable performance. Thermogravimetric analysis further demonstrated the superior thermal resistance of silane-modified wood flour, indicating improved composite durability under service conditions.

The results show that the partial replacement of PVC with renewable, carbon-storing wood flour—combined with improved interfacial adhesion—enhances the durability and mechanical integrity of the composites while reducing reliance on petrochemical raw materials. Consequently, the developed materials align with the concept of low-carbon, wood-based composites for sustainable construction. Their mechanical flexibility, resistance to degradation, and thermal stability make them suitable for use as protective and sealing layers in building envelope systems, particularly as elastic coatings for prefabricated concrete elements, interfacial layers in facade assemblies, and sealing components in panel joints and expansion zones. The materials may also serve as stress-relief and adhesion-promoting layers in prefabricated wall and facade systems, contributing to improved durability, dimensional stability, and long-term performance of energy-efficient building components.

Overall, the study demonstrates that appropriately modified wood flour enables the development of innovative, environmentally conscious PVC plastisol composites that advance durable, energy-efficient, and sustainable building technologies.

## Figures and Tables

**Table 1:** Values of temperature and maximum torque, as well as constants of Equation (1) describing the gelation process of PVC plastisol compositions with modified wood flour.

Type of WF	Modifier Content in WF, [wt%]	Temp. at Maximum Torque [°C]	Max. Torque [Nm]	Parameters of Equation (1)		
				a × 10^4^	b	c
Lignocel C-120_silane	5.0	138.0	2.75	5.30	0.19	13.70
	10.0	138.0	2.48	19.20	0.52	33.85
	15.0	137.0	1.98	13.20	0.38	25.13
	20.0	138.0	1.92	3.31	0.15	11.32
Lignocel C-120_Triton X-100	5.0	138.0	2.79	5.94	0.21	15.15
	10.0	138.0	2.39	10.60	0.32	22.07
	15.0	138.0	1.94	8.67	0.27	18.38
	20.0	138.0	1.92	8.28	0.26	18.12
Lignocel L-9_silane	5.0	128.5	3.98	43.10	1.11	67.56
	10.0	131.0	2.95	21.40	0.56	34.32
	15.0	137.0	2.78	14.20	0.39	24.47
	20.0	137.0	2.32	14.30	0.38	24.53
Lignocel L-9_Triton X-100	5.0	137.3	2.62	13.90	0.38	23.56
	10.0	138.0	2.40	8.73	0.25	15.88
	15.0	138.5	2.22	8.13	0.24	15.59
	20.0	137.0	1.97	13.10	0.35	22.42

**Table 2:** Results of density and hardness tests of PVC plastisol composite films containing modified wood flour before and after leaching.

Type of WF	Modifier Content in WF, [wt%]	Density, [g/cm^3^]	Film Hardness, [°Sh]	Weight Reduction After Leaching, [wt%]	Film Hardness After Leaching, [°Sh]
Lignocel C-120_silane	5.0	1.158	52.7	2.38	46.6
	10.0	1.158	46.0	3.35	47.0
	15.0	1.157	44.5	5.12	46.8
	20.0	1.155	40.0	6.46	42.6
Lignocel C-120 _Triton X-100	5.0	1.152	45.7	5.61	52.6
	10.0	1.146	44.6	5.83	50.6
	15.0	1.143	43.0	5.36	48.3
	20.0	1.139	40.6	5.12	46.3
Lignocel L-9_silane	5.0	1.141	77.0	4.36	57.3
	10.0	1.145	75.5	3.89	59.0
	15.0	1.143	72.0	4.27	62.0
	20.0	1.142	70.0	4.10	63.0
Lignocel L-9 _Triton X-100	5.0	1.133	74.3	6.68	61.3
	10.0	1.150	71.8	4.78	62.6
	15.0	1.152	67.3	4.37	65.3
	20.0	1.145	66.3	5.28	66.6

**Table 3:** Constants of the equation describing the dependence of the deformation of polymer-wood composites on temperature, the amount and type of wood flour, and the modifier.

Type of WF	Modifier Content in WF, [wt%]	d, e, f Parameter Values of WPC from Equation (2)		
		d	e	f
Lignocel C-120_silane	5.0	1.06	7.02	4.25
	10.0	0.11	10.23	18.00
	15.0	0.08	16.48	23.92
	20.0	0.05	22.36	32.35
Lignocel C-120_Triton X-100	5.0	1.08	14.23	6.92
	10.0	0.72	18.26	15.72
	15.0	0.66	22.41	26.40
	20.0	0.47	28.90	31.29
Lignocel L-9_silane	5.0	1.01	15.10	8.07
	10.0	0.58	20.35	17.74
	15.0	0.45	24.16	23.75
	20.0	0.37	29.33	30.67
Lignocel L-9_Triton X-100	5.0	1.04	12.49	15.87
	10.0	0.81	18.12	24.32
	15.0	0.69	24.51	31.60
	20.0	0.58	30.18	39.44

**Table 4:** Values of maximum tensile stress and relative elongation of PVC plastisol composite films containing modified wood flour before and after leaching.

Type of WF	Modifier Content in WF, [wt%]	Max. Tensile Strength, [MPa]	Relative Elongation, [%]	Max. Tensile Strength After Leaching, [MPa]	Relative Elongation After Leaching, [%]
Lignocel C-120_silane	5.0	0.86	98.1	0.82	117.3
	10.0	1.01	147.9	0.93	160.8
	15.0	1.03	175.6	1.02	183.6
	20.0	1.05	180.5	1.12	242.1
Lignocel C-120_Triton X-100	5.0	0.80	75.6	0.76	80.3
	10.0	0.78	74.3	0.74	76.1
	15.0	0.79	78.4	0.75	85.8
	20.0	0.82	83.3	0.98	110.6
Lignocel L-9_silan	5.0	0.60	16.2	0.26	27.4
	10.0	0.54	17.3	0.35	34.9
	15.0	0.56	30.0	0.38	34.8
	20.0	0.56	29.4	0.39	45.3
Lignocel L-9_Triton X-100	5.0	0.55	26.6	0.31	35.7
	10.0	0.54	25.0	0.40	34.3
	15.0	0.58	26.6	0.36	31.7
	20.0	0.56	31.8	0.43	36.4

**Table 5:** Initial, deflection, and final temperatures and corresponding values of the conservative modulus in the glass transition range.

Type of WF	Modifier Content in WF, [wt%]	Initial Temperature, [°C]	Modulus, E’ [MPa]	Bending Deflection Temperature, [°C]	Modulus, E’ [MPa],	Temperature at the End of the Glass Transition, [°C]	Modulus, E’, [MPa]
Lignocel C-120_silane	5.0	−102.9	5200	−87.4	2600	−70.1	0
	10.0	−101.2	5400	−86.1	2800	−69.5	0
	15.0	−101.3	6000	−86.4	3200	−69.7	0
	20.0	−100.0	5400	−86.8	3000	−70.4	0
Lignocel C-120_Triton X-100	5.0	−100.7	6000	−85.0	3200	−67.9	0
	10.0	−98.6	5000	−83.7	2800	−66.1	0
	15.0	−99.6	5000	−84.0	3000	−66.8	0
	20.0	−98.5	6000	−83.5	3200	−65.3	0
Lignocel L-9_silane	5.0	−100.4	4100	−83.1	2200	−63.7	100
	10.0	−98.9	4400	−82.6	2400	−62.2	200
	15.0	−99.8	4000	−83.9	2200	−65.4	200
	20.0	−99.3	4400	−83.2	2400	−64.4	200
Lignocel L-9_Triton X-100	5.0	−98.8	4000	−82.6	2200	−62.8	200
	10.0	−96.7	4200	−80.0	2600	−58.2	200
	15.0	−98.9	4400	−80.8	2600	−58.6	200
	20.0	−95.5	4000	−78.9	2400	−55.6	200

**Table 6:** Maximum temperatures corresponding to loss moduli and mechanical loss tangent.

Type of WF	Modifier Content in WF, [wt%]	Temp Max. Temperature, [°C]	Loss Tangent for Maximum E”, MPa	Temp. of the Max. Value of the Mech. Loss tangent, [°C]	Mechanical Loss Tangent, tgδ
Lignocel C-120_silane	5.0	−88.4	690.1	−56.0	0.566
	10.0	−87.8	769.0	−55.4	0.589
	15.0	−86.8	780.0	−57.5	0.570
	20.0	−88.8	796.3	−56.2	0.597
Lignocel C-120_Triton X-100	5.0	−85.8	751.4	−53.0	0.601
	10.0	−84.9	692.6	−53.7	0.566
	15.0	−84.8	764.2	−51.4	0.609
	20.0	−84.2	804.4	−53.7	0.525
Lignocel L-9_silane	5.0	−84.2	457.4	−53.7	0.377
	10.0	−83.7	510.4	−52.0	0.373
	15.0	−85.3	496.1	−55.7	0.410
	20.0	−84.5	545.5	−50.5	0.446
Lignocel L-9_Triton X-100	5.0	−84.2	480.9	−53.4	0.380
	10.0	−81.3	516.9	−45.9	0.355
	15.0	−83.3	491.7	−45.9	0.367
	20.0	−76.6	420.2	−48.1	0.350

**Table 7:** Temperature values determining the transformations occurring during the first and second stages of decomposition of composite membranes made of PVC plastisol and modified wood flour.

Type of WF	Modifier Content in WF, [wt%]	Tp1, [°C]	Tm1, [°C]	Tm2, [°C]	Tk1, [°C]	Tp2, [°C]	Tm3, [°C]	Tk2, [°C]
Lignocel C-120_silane	5.0	172.0	252.0	288.0	347.0	393.0	450.0	495.0
	10.0	175.0	255.0	292.0	349.0	397.0	456.0	512.0
	15.0	179.0	258.0	296.0	350.0	399.0	457.0	514.0
	20.0	182.0	262.0	299.0	351.0	405.0	459.0	525.0
Lignocel C-120_Triton X-100	5.0	186.0	263.0	296.0	354.0	398.0	449.0	525.0
	10.0	184.0	257.0	292.0	348.0	396.0	448.0	513.0
	15.0	179.0	252.0	287.0	344.0	394.0	446.0	507.0
	20.0	178.0	250.0	283.0	340.0	392.0	442.0	502.0
Lignocel L-9_silane	5.0	181.0	274.0	-	339.0	383.0	453.0	517.0
	10.0	183.0	275.0	-	340.0	385.0	456.0	517.0
	15.0	185.0	275.0	-	342.0	385.0	458.0	517.0
	20.0	185.0	275.0	-	342.0	388.0	460.0	517.0
Lignocel L-9_Triton X-100	5.0	184.0	278.0	295.0	338.0	381.0	452.0	514.0
	10.0	179.0	276.0	292.0	337.0	380.0	450.0	514.0
	15.0	173.0	271.0	-	334.0	376.0	449.0	506.0
	20.0	169.0	266.0	-	333.0	375.0	448.0	501.0

**Table 8:** Temperature values determining the changes occurring during the first and second stages of decomposition of composite membranes made of PVC plastisol and modified wood flour after leaching in boiling water.

Type of WF	Modifier Content in WF, [wt%]	Tp1, [°C]	Tm1, [°C]	Tk1, [°C]	Tp2, [°C]	Tm3, [°C]	Tk2, [°C]
Lignocel C-120_silane	5.0	204.0	302.0	344.0	409.0	456.0	514.0
	10.0	212.0	284.0	347.0	421.0	453.0	518.0
	15.0	216.0	277.0	345.0	427.0	464.0	517.0
	20.0	219.0	269.0	346.0	431.0	471.0	520.0
Lignocel C-120_Triton X-100	5.0	219.0	282.0	340.0	423.0	440.0	520.0
	10.0	217.0	274.0	341.0	418.0	447.0	516.0
	15.0	219.0	269.0	345.0	411.0	441.0	521.0
	20.0	215.0	265.0	344.0	402.0	444.0	518.0
Lignocel L-9_silane	5.0	226.0	295.0	344.0	404.0	470.0	509.0
	10.0	222.0	296.0	346.0	413.0	472.0	511.0
	15.0	220.0	295.0	345.0	417.0	474.0	509.0
	20.0	219.0	295.0	348.0	420.0	472.0	512.0
Lignocel L-9_Triton X-100	5.0	219.0	281.0	344.0	429.0	469.0	525.0
	10.0	224.0	278.0	341.0	425.0	471.0	524.0
	15.0	220.0	274.0	342.0	421.0	473.0	520.0
	20.0	218.0	280.0	344.0	423.0	470.0	526.0

## Data Availability

The original contributions presented in this study are included in the article. Further inquiries can be directed at the corresponding authors.
